# Comparative trial between the use of amoxicillin and amoxicillin clavulanate in the removal of third molars

**DOI:** 10.4317/medoral.19778

**Published:** 2014-06-01

**Authors:** Fernando Iglesias-Martín, Alberto García-Perla-García, Rosa Yañez-Vico, Elena Rosa, Esther Arjona-Gerveno, Juan D. González-Padilla, Jose L. Gutierrez-Pérez, Daniel Torres-Lagares

**Affiliations:** 1 Master in Oral Surgery. University of Seville (SPAIN) - Virgen del Rocío University Hospital - Seville, SPAIN

## Abstract

Objectives: The purpose of this study was to compare the use of amoxicillin (1g) vs amoxicillin and clavulanate (875/125mg) after extraction of retained third molars for prevention of infectious complications. Study Design: The study involved 546 patients attending for removal a retained third molar and divided in to two groups: Group 1 - amoxicillin and clavunate (875/125mg) group (n=257) and Group 2 - amoxicillin (1g) group (n=289). All patients were recalled for investigating the possibility of infection, presence of diarrhea and further analgesic intake. Results: From a total of 546 patients, the frequency of infection was 1.4%, without no statistically differences between the two groups. Group 1 showed statistically higher presence of patients with gastrointestinal complications (p>0.05). In 546 patients, 2.7% of patients reported severe pain that would not relieve with medication. Conclusions: The results of our study show that the use of amoxicillin (1g) and amoxicillin and clavunate (875/125mg) is similar efficacious in preventing infection after retained third molar extraction but amoxicillin and clavunate (875/125mg) produces more gastrointestinal discomfort.

** Key words:**Amoxicillin, clavulanate, third molars, complications.

## Introduction

The extraction of retained third molars is one of the most frequently procedures in common practice in dental offices and maxillofacial surgery (1). This type of intervention is classified as a “clean-contaminated surgery.” Common complications of the removal of retained third molar are pain, swelling, dysphagia and trismus. However, they are also relatively frequent infectious complications as alveolitis (2) (20-30%) and surgical wound infection (1-6%) (3). Because of these, some authors support the establishment of antibiotic guidelines are necessary to prevent from them (3). Nevertheless, antibiotic prophylaxis in third molar surgical removal remains controversial (4,5).

The aim of this study was to assess the influence of the use of clavulanate in the post-operatory after the surgical removal of retained third molar, added to the amoxicillin and, concerning to the obtained results, evaluate the viability to incorporate clavunate for prevention of infection complications.

## Patient and Methods

The study sample involved patients derived from the Virgen del Rocío University Hospital of Seville and from the Dental School at the University of Seville that fulfill the inclusion criteria: age over 18 years old, no allergy to penicillin or drugs used in this study, need of surgical removal of a retained third molar and assent of informed consent. Patients excluded were pregnant women and patients who needed more than 30 minutes time surgery. After extraction, subjects were assigned and divided in two groups. Group 1 was performed with patients who were prescribed amoxicillin and clavunate 875/125mg every 8 hours for 7 days. Patients who were prescribed amoxicillin 1g every 8 hours for 7 days make the Group 2. Finally, we conducted a randomized trial in 546 patients (Group 1: 257 patients; Group 2: 289 patients) requiring surgical removal of included third molars. The groups were comparable from the standpoint of position, orientation, impaction and history of pericoronitis. The investigation was previously approved by the Ethical Committee of the University Hospital.

After surgery, postoperative instructions were explained carefully to all patients and they were only prescribed anti-inflammatory and analgesic medication (ibuprofen 600mg every 8 hours at the most) for postoperative pain relief. Oral and written recommendations were given.

On the sixth day patients were request by telephone about their condition. Registered variables were:

-Pus. Patients were asked about the presence of purulent liquid through the wound or severe halitosis.

-Fever above 38°C after the first 48 hours 

-Pain and relief of pain with anti-inflammatories and painkillers, which indicated the possibility of clinical diagnosis of alveolitis (2).

-Inflammation persistent over time that does not improve during the week.

To integrate this different criterion, the infection criterion used in this study is the presence of pus with another positive criterion (of previously mentioned) or three positive criterion (of previously mentioned).

-Lockjaw or trismus. This criterion was evaluated as the inability of the patient to introduce two fingers transversally in their mouth.

-Gastrointestinal upset.

A complete clinical examination and checkup about previous described variables was performed on seventh day in patients who had any of these first five criteria, to confirm the presence of infection.

All data collected were analyzed using the SPSS statistical package. Comparisons between groups were analyzed using the chi-square test. Statistically differences were considered at *p*>0.05

## Results

The study involved 546 patients, 233 men and 313 women. The average age of males was 29.04 years and women 27.93 years. In Group 1, 257 patients (29.12+10.09 years; 96 males; 161 females) were included and in the Group 2, 289 (28.09+8.24 years; 137 males; 152 females) patients were studied. All patients received a full antibiotic treatment as previously was described.

Most of variables studied showed higher frequency in amoxicillin (1g) patients (Group 2), except the presence of fever above 38°C after the first 48 hours and complaint of gastrointestinal upset ( Table 1).

**Table 1:**
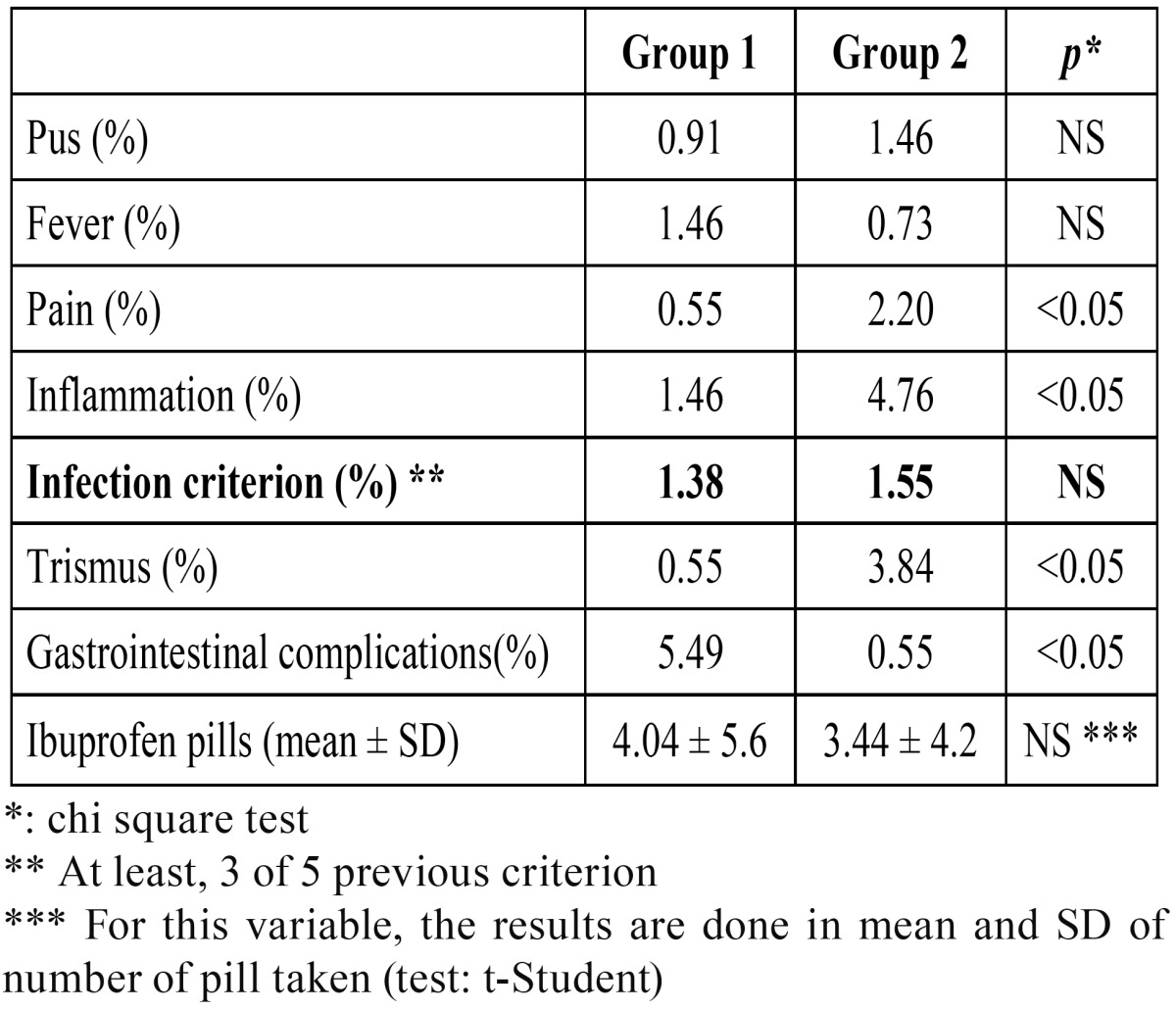
Comparison of presence of registered variables (%) and number of ibuprofen pills ((mean ± SD) in amoxicillin and clavulanate 875/125mg (group 1) and amoxicillin 1g (group 2) patients.

Chi square test was used to compare postoperative variables between patients who used amoxicillin (1g) vs amoxicillin and clavulanate (875/125mg) after extraction of third molars. No statistically significant differences (*p*>0.05) were found in the presence of pus, fever, and relief of pain with other drugs. Statistically differences were found (*p*<0.05), in the presence of alveolitis, per-sistence of inflammation, trismus and gastrointestinal complications ( Table 1). However, we found no statistically significant differences (*p*>0.05) in the presence of criterion of infection between the two groups.

## Discussion

The use of antibiotic prophylaxis is usually in third molar surgery since it is considered clean-contaminated surgery. However the use and the correct use of routine antibiotic prophylaxis is a controversial topic (6). Infectious pathology associated with third molars has created the necessity of multiple studies that have been carried out with different antibiotics, and whether infection prevention is really necessary or not (7). There is no consensus on the antibiotic of choice for prophylaxis guidelines (8). Siddigi’s and Zeitler’s research have shown that the infection of the surgical wound infection rate is low and infrequent, then they do not advise the use of antibiotics as routine prevention in the removal of third molars (3,9).

However, many authors suggest that there are significant differences of infection in groups of patients who are treated with antibiotic therapy compared to those who received placebo (10-13). In fact, the results of the well-done metanalisis by Fang Yang revealed that the use of antibiotics improves postoperative complaints of the patients and reduces the appearance of infection (13).

The most frequently isolated bacteria in odontogenic infection are Streptococcus viridans, Peptoestreptococo, Prevotella intermedia, Fusobacterium nucleatum and Porphiromona gingivalis (14,15). Kuriyama noted in their studies that 7% of these species produced beta-lactamase, and that production was associated with the previous use of beta-lactam antibiotics (16). In order to reduce bacterial resistance, clavulanate became associated with amoxicillin (17). Some researchers have found that clindamycin and amoxicillin with clavulanate are the most effective antibiotics in established odontogenic infection (15).

There is no consensus on the use of antibiotics in the extraction of retained third molars, and neither the type of antibiotic of choice in case of support it (18-20). Our purpose was to study the selection of the correct antibiotic for the prevention of infection after the surgical removal of third molars as one of the principles of antibiotic prophylaxis delineated by Peterson (21). In other words, the use or not of clavulanate with amoxicillin. We followed-up 546 patients with surgical removal of a retained third molar during 7 days.

In our study we found that amoxicillin produced more swelling, trismus and pain (although without statistically significant differences) but significant less gastrointestinal discomfort that the combination with clavulante. However, amoxicillin (1g) and amoxicillin/clavulanate (875/125mg) were equally effective in preventing infection after third molar extraction.

Though there is true that all the patients were not examined and the post-operative screening assessment was accomplished by phone greatly, and this detracts from the value of the study (for example, it is well know that a patient can return with occult purulent drainage that will only be discovered with a careful examination), the data are so clear that the conclusions are acceptable.

Our results are in concordance with the literature who studied the same parameters in the prevention of infection after the removal of retained third molars. The pharmacovigilance group of Italy noted that clavulanate had a higher number of complications especially in the gastrointestinal system, and even bacteria can also create resistance to clavulanate. They conclude that amoxicillin is the antibiotic of choice except in patients with severe infections (22). Bresco’s studies concluded that the most effective antibiotic in the treatment of odontogenic infection was amoxicillin with clavulanate but clinical effects are very similar, so it does not make any difference between their use (15).

To sum up and after analyzed our results, if there is no difference in the prevention of infection after surgical removal of retained third molars with amoxicillin of amoxicillin/clavulanate and the last one produces significant more gastrointestinal discomfort, we conclude that the association between clavulanate and amoxicillin is not indicated as routine in guidelines after extraction of third molars.
